# Laser-Ablative Synthesis of Silicon–Iron Composite Nanoparticles for Theranostic Applications

**DOI:** 10.3390/nano13152256

**Published:** 2023-08-05

**Authors:** Alexander A. Bubnov, Vladimir S. Belov, Yulia V. Kargina, Gleb V. Tikhonowski, Anton A. Popov, Alexander Yu. Kharin, Mikhail V. Shestakov, Alexander M. Perepukhov, Alexander V. Syuy, Valentyn S. Volkov, Vladimir V. Khovaylo, Sergey M. Klimentov, Andrei V. Kabashin, Victor Yu. Timoshenko

**Affiliations:** 1 Institute of Engineering Physics for Biomedicine (PhysBio), National Nuclear Research University MEPhI, 115409 Moscow, Russia; bubnovmeph@gmail.com (A.A.B.); vsbelov@mephi.ru (V.S.B.); juliakargina@gmail.com (Y.V.K.); gtikhonowski@gmail.com (G.V.T.); aapopov1@mephi.ru (A.A.P.); aykharin@mephi.ru (A.Y.K.); mvshestakov@rgau-msha.ru (M.V.S.); smklimentov@mephi.ru (S.M.K.); 2 Endocrinology Research Centre, Dmitry Ulyanov Street 11, 292236 Moscow, Russia; 3 Faculty of Physics, Lomonosov Moscow State University, Leninskie Gory 1, 119991 Moscow, Russia; 4 Moscow Timiryazev Agricultural Academy - Russian State Agrarian University, 127434 Moscow, Russia; 5 Moscow Institute of Physics and Technology, Dolgoprudny, 141700 Moscow Region, Russia; aleksandr-iv@mail.ru (A.M.P.); alsyuy271@gmail.com (A.V.S.); vsv.mipt@gmail.com (V.S.V.); 6 Department of Functional Nanosystems and High-Temperature Materials, National University of Science and Technology MISIS, Leninskiy Prospekt 4, 119049 Moscow, Russia; khovaylo@misis.ru; 7 LP3, Aix Marseille University, CNRS, Campus de Luminy, Case 917, 13288 Marseille, France

**Keywords:** laser ablation in liquids, composite nanoparticles, photothermal therapy, magnetic resonance imaging, silicon, iron silicide, theranostics

## Abstract

The combination of photothermal and magnetic functionalities in one biocompatible nanoformulation forms an attractive basis for developing multifunctional agents for biomedical theranostics. Here, we report the fabrication of silicon–iron (Si-Fe) composite nanoparticles (NPs) for theranostic applications by using a method of femtosecond laser ablation in acetone from a mixed target combining silicon and iron. The NPs were then transferred to water for subsequent biological use. From structural analyses, it was shown that the formed Si-Fe NPs have a spherical shape and sizes ranging from 5 to 150 nm, with the presence of two characteristic maxima around 20 nm and 90 nm in the size distribution. They are mostly composed of silicon with the presence of a significant iron silicide content and iron oxide inclusions. Our studies also show that the NPs exhibit magnetic properties due to the presence of iron ions in their composition, which makes the formation of contrast in magnetic resonance imaging (MRI) possible, as it is verified by magnetic resonance relaxometry at the proton resonance frequency. In addition, the Si-Fe NPs are characterized by strong optical absorption in the window of relative transparency of bio-tissue (650–950 nm). Benefiting from such absorption, the Si-Fe NPs provide strong photoheating in their aqueous suspensions under continuous wave laser excitation at 808 nm. The NP-induced photoheating is described by a photothermal conversion efficiency of 33–42%, which is approximately 3.0–3.3 times larger than that for pure laser-synthesized Si NPs, and it is explained by the presence of iron silicide in the NP composition. Combining the strong photothermal effect and MRI functionality, the synthesized Si-Fe NPs promise a major advancement of modalities for cancer theranostics, including MRI-guided photothermal therapy and surgery.

## 1. Introduction

To improve the quality of healthcare and advance toward highly selective personal medicine, novel efficient and minimally invasive biomedical approaches that could be promptly translated into clinical practice are still required. Methods of nanotechnology could satisfy such a demand as they offer novel advanced tools for diagnostics and therapy, especially in the field of cancer [1,2,3]. Indeed, newly emerging nanomaterials can not only provide a mechanism for the passive targeting of tumors based on the enhanced permeability and retention (EPR) effect [4], but also offer a number of functionalities for imaging or therapy based on the intrinsic physicochemical properties of these nanomaterials. As an example, the imaging functionality can arise from the photoluminescent [5,6], absorption/scattering [7], photoacoustic [2] or magnetic [8] responses of nanoparticles (NPs). The therapeutic functionality of NPs can arise from their ability to sensitize the effect of local heat release under external stimuli, e.g., photo [3] or magnetic field [9] excitation, which can be used for local hyperthermia of cancer cells, leading to their death.

Nanomaterials combining high biocompatibility and bioimaging and/or therapy potentials are of particular interest for biomedical tasks. Here, nanostructured silicon (nano-Si) occupies a unique niche, as it is not only biocompatible, but also biodegradable. Indeed, in biological tissues nano-Si dissolves into orthosilicic acid; then, products of its dissolution are safely excreted from the organism via renal clearance [10,11]. In addition, being group IV semiconductors, Si-based NPs can exhibit a series of unique imaging and therapy functionalities, including room-temperature photoluminescence [5] and non-linear optical response [12] for bioimaging, light-induced generation of singlet oxygen for photodynamic therapy [13], infrared irradiation-induced [14,15] and radiofrequency-radiation-induced [16] hyperthermia for cancer therapy. Iron (Fe)-based nanostructures represent another promising nanomaterial for biomedical applications [17]. Iron oxide NPs are also biocompatible and biodegradable, although their use in vivo has some tolerance limits due to possible residual toxicity of decaying Fe-based compounds [18]. It is important that iron oxide NPs exhibit prominent physical properties, which makes them an ideal candidate for both magnetic resonance imaging (MRI) [8,19] and magnetic-hyperthermia-induced therapy [20,21].

The combination of silicon- and iron-based compounds in a single nanoformulation presents a promising approach to developing a dual-modal magnetic-semiconductor nanoplatform for cancer theranostics. In this case, one could profit from a combined signal of Si and Fe components, e.g., to simultaneously enable efficient MRI contrast and photothermal therapy options [21,22]. However, there are severe limitations in the fabrication of Si-Fe structures suitable for biomedical use. Conventional methods for the synthesis of such structures are based on wet chemistry routes, but these methods typically involve non-biocompatible chemicals and toxic by-products, which drastically complicates the use of synthesized nanomaterials in biological systems [23]. Alternative pathways are based on methods of “dry” plasma synthesis [24,25]. In particular, we recently reported the fabrication of Si-Fe composite NPs with a variable content of Fe by an arc-discharge plasma-ablative method. Polycrystalline Si powder mixed with 0.2–10% Fe was evaporated at 5500 °C in an arc-discharge reactor, and then cooled down to form NPs [24,25]. Our tests also showed that the NPs with the content of Fe exceeding 2.5% could provide a strong shortening of both the longitudinal and transverse proton relaxation times in magnetization relaxometry, which made the achievement of high MRI contrast possible in experiments in vivo [25]. However, an increase in toxicity was observed when the concentration of nanoparticles was larger than 50 μg/mL [25,26], which can complicate the biological prospects of these NPs [26].

Here, we explore the use of methods of pulsed laser ablation in liquids (PLAL) to engineer Si-Fe NPs, which could combine magnetic and photothermal functionalities. Based on a natural formation of nanoclusters during the action of laser radiation on a solid target, PLAL profits from clean and essentially non-equilibrium conditions of nanostructure growth, which makes the formation of non-toxic complex multi-component nanoformulations possible [27]. As we have shown in previous studies, owing to the much lower amount of energy required to ablate a unity of material, femtosecond (fs) laser ablation is especially efficient in controlling the size characteristics of formed nanomaterials, from single spherical NPs [28,29,30] to complex multi-functional core-satellite or core-shell nanoarchitectures [31,32,33]. The choice of this technique for the engineering of Si-Fe nanoformulations is justified by specific nanofabrication conditions: (i) the easy preparation of different compositions of NPs, which can be obtained only in the form of colloidal solutions; (ii) the nearly spherical shape of Si-Fe NPs (iii) the inherent stability of prepared colloidal solutions of bare (ligand-free) NPs and (iv) the possibility of the minimization of residual toxic by-products.

In this study, the fs laser technique of PLAL was applied to ablate a composite target fabricated from hot-pressed powders of silicon and iron silicide. Physicochemical and photothermal properties of the obtained NPs were thoroughly analyzed. The obtained results demonstrate that the prepared silicon–iron composite NPs are very promising for the projected multimodal theranostic applications.

## 2. Materials and Methods

### 2.1. Materials

Silicon (Si, 99.998%, CAS No. 7440-21-3) and iron (Fe, 99.99%, CAS No. 7439-89-6) polycrystalline powders, as well as acetone (CH3COCH3, 99.9%, CAS No. 67-64-1), were purchased from Sigma-Aldrich (St. Louis, MO, USA). Mili-Q water was used as a solvent.

### 2.2. NP Synthesis

Targets were prepared by using spark plasma sintering (SPS) of mixed polycrystalline microgranular powders of high-purity silicon and iron in the atomic ratio of 2:1 using a Labox 650 (Sinter Land Inc., Kyoto, Japan) apparatus.

Si-Fe NPs were synthesized using methods of femtosecond laser ablation of the prepared targets immersed in acetone, according to the procedure described in previous studies [34]. Briefly, the composite target was vertically fixed in a quartz cuvette at 3 mm from the side wall. The laser beam was focused through the side wall on the surface of the target using a 100 mm working distance F-theta objective. The ablation was conducted using a femtosecond Yb: KGW laser Teta 10 (Avesta, Moscow, Russia) with a wavelength of 1030 nm, pulse repetition rate of 100 kHz and pulse energy of 30 μJ. The laser beam scanned the surface of the target at 5 m/s using a galvanometric scanner. The cuvette was filled with 60 mL of acetone. The synthesis duration was 30 min. The concentration of Si-Fe NPs in the colloidal solution was estimated as 1.2 mg/mL, which was determined by weighing the target before and after synthesis.

For further studies, Si-Fe NPs were transferred from acetone to water by centrifugation (Microspin 12, BioSan, Riga, Latvia) at a relative centrifugal force of 5890× *g* for 10 min, followed by mixing with de-ionized water to obtain the NP concentration of approximately 1 mg/mL. Additionally, pure Si NPs were synthesized by femtosecond ablation of a crystalline Si target in water.

### 2.3. NP Characterization

The size, morphology and semi-quantitative chemical composition of formed NPs were examined by transmission electron microscopy (TEM) using a Jeol JEM-2100 microscope (JEOL Ltd., Tokyo, Japan) operating at 200 kV. The chemical composition was characterized by an energy-dispersive spectrometry (EDS) detector X-act, Aztech X-Max 100 (Oxford Instruments, High Wycombe, UK), attached to the TEM system. Samples were prepared by dropping 20 μL of the Si-Fe NPs solution onto a Cu grid, which was covered by a carbon film, and subsequent drying under ambient conditions. The size (diameter) distribution of NPs was plotted after measuring at least 250 NPs using ImageJ software (version 1.53t).

Hydrodynamic sizes and ζ-potentials were measured for 10-fold diluted aqueous colloids of Si-Fe NPs by using a Zetasizer Nano ZS (Malvern Instruments, Malvern, UK).

Raman spectra of dried Si-Fe NPs deposited on stainless steel foil were recorded under a 633 nm laser excitation with 1 mW power using a Confotec MR350 micro-Raman spectrometer (SOL Instruments, Minsk, Belarus). The laser beam was focused with a 50× microscope objective, and the acquisition time was 30 s. The measurements were carried out under ambient conditions. Special attention was given to temperature control in order to prevent overheating of the samples during measurements.

An X-ray diffraction (XRD) study was performed by using a Radian (NTC Expert center, Moscow, Russia) apparatus with a Cu Kα line (λ = 0.154184 nm) under ambient conditions.

Electron paramagnetic resonance (EPR) spectra of dried Si-Fe NPs were measured at room temperature by using a X-band EPR spectrometer CMS 8400 Adani (Linev Systems Inc., Conroe, TX, USA), with the center frequency of a rectangular TE102 cavity of f = 9.4 GHz and magnetic field of B ≤ 0.7 T. Effective g-factors of the samples were calculated with respect to a reference sample of a,g-bisdiphenyline-b-phenylallyl (BDPA) with the g-factor of 2.00359.

Photothermal properties were evaluated by irradiating aqueous solutions of Si-Fe and Si NPs (1 mL, 1 mg/mL) by a CW semiconductor laser operating at 808 nm with the power of 384 mW under ambient conditions. The collimated laser beam was 2 mm in diameter and the optical path length of a cuvette was 10 mm. Temperature was measured by a platinum thermometer with an accuracy of 0.01 °C and a 15 Hz rate. To avoid inhomogeneous photoheating, we used a magnetic stirrer with a frequency of 110 rpm, which allowed us to condition a uniform temperature of the whole volume during the recording of heating–cooling transitions. The photothermal conversion efficiency was determined from the temperature transitions according to an analysis proposed in Refs. [35,36].

Optical extinction spectra were recorded in the range from 350 to 1000 nm using a UV752P spectrophotometer (Wincom Ltd., Changsha, China) in quartz cuvettes with a 10 mm optical path length. The optical measurements were carried out for aqueous suspension of Si-Fe and Si NPs with a 0.1 mg/mL concentration.

Longitudinal and transverse proton relaxations were investigated by a Minispec NMR (Bruker, Billerica, MA, USA) relaxometer equipped with a 20 MHz probe, at 40 °C.

## 3. Results

### 3.1. Size and Morphology of NPs

**Figure 1:**
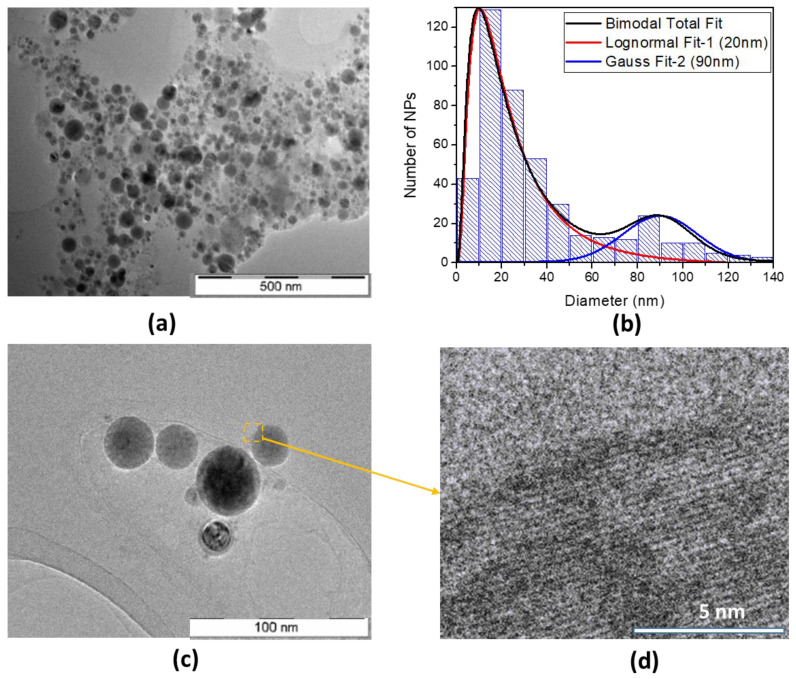
TEM characterization of the laser-synthesized Si-Fe NPs. (**a**) Typical large-view TEM image of NPs; (**b**) Size distribution of NPs obtained from TEM images. The size distribution can be extrapolated by a bimodal Gaussian function fit (red line) as a total fit. In addition, Gaussian function fits can be applied to separately extrapolate 20 nm (green line) and 90 nm (black line) fits; (**c**) TEM image of a group of NPs; (**d**) High-resolution TEM image of a selected region of the Si-Fe NP marked in panel (**c**).

Size and morphology of the synthesized NPs were analyzed by electron microscopy. A typical TEM image (Figure 1a) shows a conglomerate of dried Si-Fe NPs. The observed aggregation of the NPs is caused by the sample preparation technique and does not represent their colloidal properties. NPs with different transparencies for electron beams are clearly observable, suggesting that the synthesized nanocomposites do not have a uniform distribution of Si and Fe.

The results of the statistical analysis of TEM images are shown in Figure 1b. One can see that the laser-synthesized Si-Fe NPs exhibit a broad size distribution from 10 to 150 nm. The size distribution can be fitted by bimodal Gaussian and log-normal functions: for populations with mean sizes of 20 and 90 nm, these are log-normal and Gaussian function fits, respectively. The size distribution of Si NPs can also be fitted by a log-normal function (Figure S1) with the mean size of 30 nm. One can see from Figure 1c and Figure S1 that all laser-synthesized NPs are mostly spherical. A high-resolution TEM imaging (Figure 1d) demonstrates that the synthesized NPs have both nanocrystalline and amorphous areas (dark area inside NPs in Figure 1c). The coexistence of amorphous and nanocrystalline phases in prepared Si-Fe NPs can be related to the non-equilibrium conditions of PLAL when the fast cooling does not allow complete crystallization. The latter is also dependent on the composition of NPs. As for the size distribution, we suggest that the two populations could appear due to the phenomenon of the metal-stimulated growth of Si NPs at iron-rich surface regions. This effect has already been reported for similar Si-Fe NP formed by dry plasma-ablative synthesis in Ar plasma jets [25].

### 3.2. Composition and Structure of NPs

**Figure 2:**
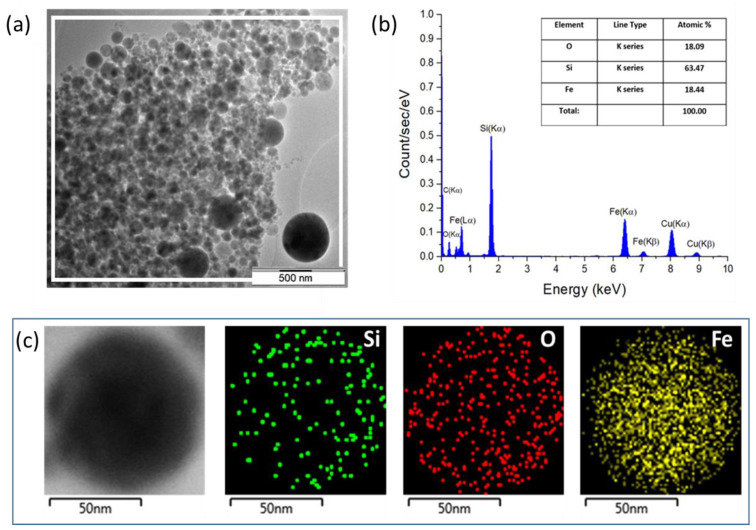
EDS characterization of Si-Fe NPs. (**a**) TEM image of Si-Fe NPs, in which a white square border indicates the region selected for EDS analysis; (**b**) EDS spectrum and atomic composition of the NPs shown in panel (**a**); (**c**) Images of a Si-Fe NP obtained in a combined TEM and EDS mode for Si, O and Fe.

The chemical composition of the Si-Fe NPs was measured by the EDS technique. An area containing a large number of NPs (Figure 2a) was analyzed to obtain an averaged composition of NPs. As is shown in Figure 2b, the EDS spectrum of the Si-Fe NPs consists of characteristic Fe lines, including K_α_ (6.404 keV), K_β_ (7.058 keV), L_α_ (0.703 keV) and L_β_ (0.525 keV). In addition, the spectrum contains a characteristic K_α_ (0.525 keV) line of oxygen and K_α_ (1.74 keV) line of Si. In general, this spectrum illustrates a dominant contribution of signals from Si, Fe and O with and atomic % of 64, 18 and 18, respectively. Additional EDS peaks in Figure 2b can be attributed to contributions of carbon and copper from the TEM grid.

The spatial distribution of silicon, iron and oxygen atoms within a Si-Fe NP is shown in Figure 2c. Here, one can see the homogeneous distribution of all elements in the composite NP. The obtained composite is likely composed of fractions of nanocrystalline silicon, iron silicide, amorphous silicon and iron oxides.

**Figure 3:**
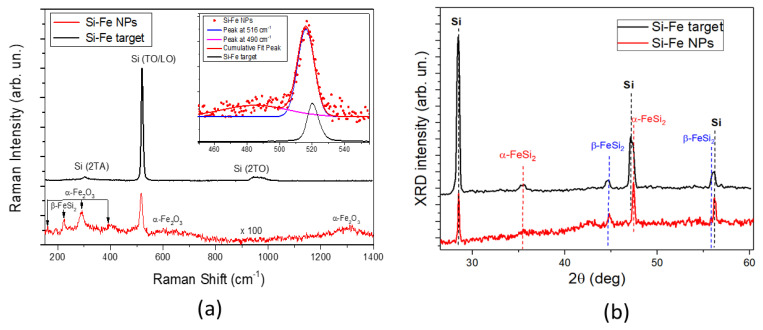
(**a**) Raman spectra from dried Si-Fe NPs and Si-Fe target used. The inset shows the fitting results in the region of 450–550 cm^−1^; (**b**) XRD spectra from dried Si-Fe NPs and Si-Fe target.

Figure 3a shows the results of the structural characterization of dried Si-Fe NPs and that of the ablation target by Raman spectroscopy. One can see that the Raman signal from the target only has peaks associated with the crystalline Si lattice, while other phases related to iron silicide or iron oxides cannot be detected. On the other hand, the presented Raman spectrum from Si-Fe NPs has more features, as it contains a sharp peak at 516 cm^−1^ and a broad band centered at 490 cm^−1^, which are attributed to the Si nanocrystalline and amorphous Si phases, respectively [37]. Note that the recorded shift in Raman peak of crystalline Si from 520 cm^−1^ to 516 cm^−1^ (Figure 3b) for the spectra obtained from the ablation target and Si-Fe NPs, respectively, indicates that Si nanocrystals have sizes of a few nanometers, while the size of NPs is tens of nanometers. Therefore, laser-ablated Si-Fe NPs have a polycrystalline structure. Additional peaks in the Raman spectrum of NPs can be attributed to the iron oxide (Fe_2_O_3_) and iron silicide (β-FeSi_2_) [38,39].

The XRD spectra from Si-Fe NPs and the Si-Fe target are shown in Figure 3b. One can easily find that the main peaks in the spectrum from the target correspond to the crystalline Si lattice, while weak peaks at diffraction angles of 35.22 and 44.45 deg. can be attributed to two phases of iron silicide (α-FeSi_2_ and β-FeSi_2_). The peak near 56 deg. could be associated with either Si or β-FeSi_2_. The spectrum of Si-Fe NPs contains similar peaks, but in this case, a well resolvable peak of α-FeSi_2_ at 35.22 deg is absent. It should also be noted that the XRD technique does not reveal the presence of crystalline iron oxides, which means that the iron oxides are mostly in an amorphous phase.

### 3.3. Optical and Photothermal Properties

**Figure 4:**
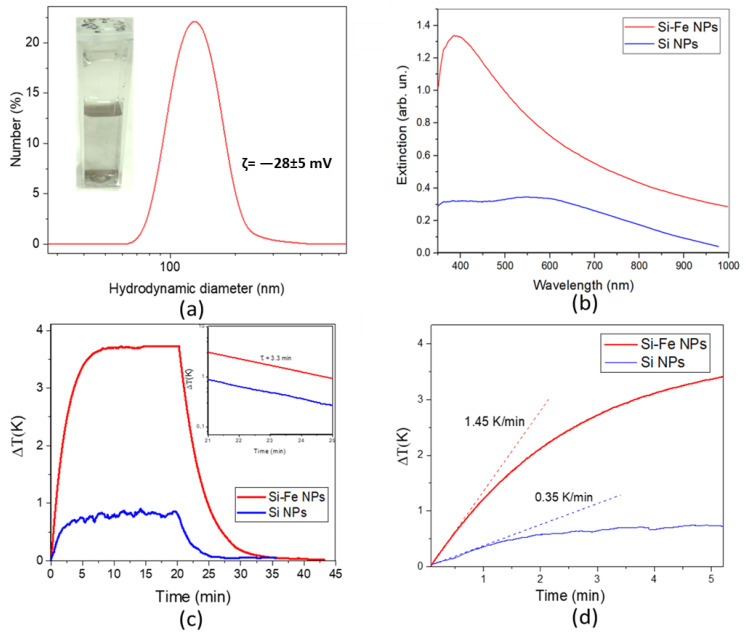
Optical and photothermal properties of Si-Fe and Si NPs. (**a**) DLS size distribution of Si-Fe NPs in an aqueous suspension. The inset shows a photographic image of a vessel filled with a Si-Fe NP suspension in water; (**b**) Extinction spectra from 0.1 mg/mL solutions of NPs; (**c**) Transitions of the photoheating and cooling after switching off the laser irradiation for aqueous suspensions of Si-Fe (red curve) and Si (blue curve) NPs at concentration of 1 mg/mL. The inset shows the cooling kinetics at a semilogarithmic scale. (**d**) Initial parts of the photoheating kinetics for aqueous suspensions of Si-Fe (red curve) and Si (blue curve) NPs at concentration of 1 mg/mL.

Figure 4a shows a typical DLS spectrum from Si-Fe NPs in an aqueous suspension and an image of the suspension in a plastic cuvette (inset). The mean hydrodynamic diameter of the NPs is approximately 100 nm. A slightly larger value measured by DLS compared to the physical size (measured by TEM) can be explained by the formation of a hydration layer near the NP’s surface. Moreover, the sensitivity of the DLS technique is highly size-dependent and a small fraction of large NPs can completely mask more-abundant smaller NPs. The large hydrodynamic size can also originate from the aggregation of NPs, but this supposition is not confirmed by a high stability of the NP’s size during their storage under ambient conditions for several months. The high colloidal stability of the laser-synthesized NPs is obviously due to an electrostatic stabilization as a result of charging of their surface during the synthesis. This supposition is confirmed by a high value of the ζ-potential (–28 ± 5 mV), which exceeds the stability threshold at room temperature.

Optical extinction spectra from the colloidal solutions in the visible–near-IR range are shown in Figure 4b. One can see that the spectrum from Si-Fe NPs has a peak near 400 nm with a long tail extending to the IR, while the spectrum from Si NPs has a broad peak centered near 540 nm, which could be attributed to the activation of dielectric Mie resonances in Si NPs. Optical extinction is higher for Si-Fe NPs than for Si NPs in the whole measured range. Photo heating–cooling curves for both NP types under the irradiation of an 808 nm laser are shown in Figure 4c. The maximal achievable temperature increment for the Si-Fe NPs was approximately four-fold higher than that for Si NPs (3.75 ± 0.05 and 0.85 ± 0.05 K, respectively). Both types of NP demonstrated a high photostability under near-IR irradiation and did not change their photothermal properties in multiple heating–cooling cycles.

To evaluate the photothermal conversion efficiency of Si-Fe NPs, we analyzed the temperature transitions during and after the photoexcitation (Figure 4c). It was assumed that water is nearly transparent for the employed IR laser radiation and the mass fraction of NPs in the studied solutions with an NP concentration of 1 mg/mL was very small, i.e., 0.1%. Then, the temperature change in a solution with dispersed NPs can be described by the following thermal balance equation [36]:

(1) $$C_{w}\cdot m_{w}\cdot \frac{dT}{dt}=Q_{in}-Q_{out}\;,\;$$

where *m_w_* and *C_w_* are the mass and specific heat capacity of the photoheated solution, respectively, $\frac{dT}{dt}$ is the rate of temperature change vs. time, $Q_{in}$ is the input heat energy during photoexcitation and $Q_{out}$ is the heat dissipation from the solution to the external environment.

By neglecting the light absorbance and reflectance by water and walls of the plastic cuvette, the input heat energy during photoexcitation can be expressed by the following way [36]:

(2) $$Q_{in}=\left(I_{0}-I_{t}\right)\cdot \eta ,$$

where $I_{0}$ is the incident laser power, $I_{t}$ is the laser power transmitted through the solution of NPs and η is the photothermal conversion efficiency of NPs.

At the same time, the heat dissipated to the external environment can be described by the following expression [36]:

(3) $$Q_{out}=\frac{C_{w}\cdot m_{w}\cdot \Delta T}{\tau },$$

where *τ* is the cooling time due to the heat dissipation to the external environment and ΔT is the temperature growth relative to the initial temperature.

The value of η can be obtained from Equations (2) and (3) under steady-state conditions, i.e., $Q_{in}-Q_{out}=0$, and it can be expressed as follows:

(4) $$\eta =\frac{C_{w}\cdot m_{w}\cdot \Delta T_{max}}{\left(I_{0}-I_{t}\right)\cdot \tau }\;,$$

where $\Delta T_{max}$ is the maximal temperature growth.

Figure 4c shows that the cooling decay can be fitted by a single exponential function with the decay time of approximately τ= 3.3 ± 0.1 min for both Si-Fe and Si NPs. This fact indicates a negligible effect of dispersed NPs on the heat dissipation under our experimental conditions.

By using Equation (4) to treat the results shown in Figure 4d, the photothermal conversion efficiencies for Si-Fe and Si NPs reach 33 ± 5% and 10 ± 3%, respectively.

The initial photoheating rate of the solution with Si-Fe NPs is approximately 1.45 ± 0.06 K/min and it is only 0.35 ± 0.05 K/min for Si NPs, as shown in Figure 4d. A higher heating rate of the former NPs agrees with their larger photothermal efficiency. Note that the initial photoheating rate can be used to estimate the photothermal conversion by NPs. Indeed, the initial growth of temperature during photoexcitation can be expressed from Equation (1) in the following way:

(5) $$\frac{dT}{dt}\approx \frac{\left(I_{0}-I_{t}\right)\cdot \eta }{C_{w}\cdot m_{w}}\;$$

The photothermal conversion efficiencies estimated via Equation (5) by using the initial temperature growth were found to reach approximately 42 ± 4 and 14 ± 2% for Si-Fe and Si NPs, respectively. These values are close to the estimations obtained from dependences of maximal temperature growth by using Equation (4). The observed difference between values of η obtained by using the analysis of initial and steady-state parts of temperature transitions can be explained by both an uncertainty in the determination of the initial time interval and a possible contribution of additional heat losses, e.g., evaporation and air conversion flows, which can affect the maximal temperature during prolonged photoheating.

The revealed high efficiency of the photothermal conversion of Si-Fe NPs under laser irradiation at 808 nm, which is 3.0–3.3 times larger than that of Si NPs, is obviously determined by both their high optical absorbance and a relatively low light scattering efficiency of Si-Fe NPs in comparison with pure Si NPs.

### 3.4. MRI Contrasting Properties

**Figure 5:**
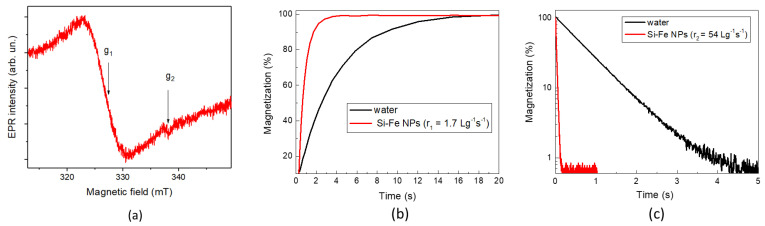
(**a**) EPR spectrum of dried Si-Fe NPs; (**b**) Transitions of the longitudinal magnetization of aqueous suspensions of Si-Fe NPs (1 mg/mL) and water, for comparison; (**c**) Transitions of the transverse proton magnetization for aqueous suspensions of Si-Fe NPs (1 mg/mL) and water, for comparison.

As is shown in Figure 5a, the EPR spectrum of Si-Fe NPs exhibits two resonance modes. The dominant broad mode with a g-factor of approximately g_1_ ≈ 2.00 can be related to Fe^3+^ ions in the iron oxide inclusions [40]. A narrow central mode of the EPR spectrum is characterized by g_2_ = 2.0055 and can be attributed to silicon dangling bonds at the Si/SiO_2_ interface [41,42]. Using a reference sample, the density of paramagnetic centers in the dried Si-Fe NPs is estimated to be 10^17^ spin/g.

A large number of paramagnetic centers in Si-Fe NPs leads to the effect of a spin–spin relaxation, which results in a shortening of proton magnetization times in aqueous suspensions of those NPs. Figure 5b,c shows transitions of the proton magnetization of pure water and aqueous suspensions of Si-Fe NPs. One can see that both the longitudinal (Figure 5b) and transverse proton magnetization (Figure 5c) become faster in the presence of Si-Fe NPs. Such data allow us to estimate a specific relaxation rate, i.e., relaxivity, which can be calculated according to the following expression:

(6) $$r_{1,2}=R_{1,2}/C,\;$$

where *C* is the mass concentration of NPs in aqueous suspension, and *R*_1_ and *R*_2_ are the relaxation rates for the longitudinal and transverse proton magnetizations, respectively, related to NPs.

Equation (5) allows us to estimate the longitudinal relaxivity of *r*_1_ = 1.7 g^−1^s^−1^L for the prepared Si-Fe NPs in aqueous suspension, while the transverse relaxivity demonstrates an even more remarkable value of *r*_2_ = 54 g^−1^s^−1^L. The observed predominant shortening of the transverse relaxation time is typical for NPs, as it was earlier reported for Si-Fe NPs obtained by plasma-ablative synthesis [24,25]. Note that the corresponding values for laser-ablated pure Si NPs are *r*_1_ = 0.2 g^−1^s^−1^L and *r*_2_ = 1.4 g^−1^s^−1^L, which indicate the major role of iron ions in the shortening the proton relaxation times.

## 4. Discussion

Thus, we showed that the femtosecond laser-ablative technique can be used to fabricate non-agglomerated spherical Si-Fe NPs with two size populations, with a mean size of 20 nm and 90 nm. The NP population with a mean size of 20 nm looks especially promising for projected biomedical applications. Indeed, this size provides a sufficiently large surface area for functionalization by biopolymers and targeting molecules. In addition, Si-Fe NPs of this size can still profit from efficient cell internalization via endocytosis mechanisms, including mechanisms of clathrin-mediated and caveolin-mediated endocytosis. On the other hand, large NPs (100 nm in size or more) can be also useful for biomedical applications, although they cannot internalize as efficiently into cells via mechanisms of receptor-mediated endocytosis and they have a reduced blood circulation time due to the increased elimination rate of large NPs by the mononuclear phagocyte system.

The prepared Si-Fe NPs are partially oxidized, which is typical for nanomaterials prepared by PLAL synthesis [29,34]. The oxidation of Si-based NPs can potentially occur at different stages during the synthesis [27]. Laser ablation in liquids is a complex process, which still does not have complete theoretical description. However, it is widely accepted that this process starts from energy absorption by the target material, followed by a material ejection in the form of hot plasma consisting of individual atoms and the smallest clusters. The energy exchange between the laser-induced plasma and surrounding liquid leads to the formation of a so-called cavitation bubble, which contains liquid molecules, products of their decomposition and dissolved gases, along with target material ions and clusters. NPs start to grow from the formed nanoclusters inside the bubble; therefore, dissolved molecular oxygen and oxygen from the decomposed liquid molecules can potentially oxidize the forming NPs. The cavitation bubble stage is followed by a slow growth and a surface oxidation at steady-state physical and chemical conditions [27]. The transformation of Si to SiO_2_ has a negative effect on the optical and photothermal properties for projected biomedical applications. Therefore, we minimized the oxidation phenomena by performing the synthesis in acetone. This strategy has already proved its efficiency in decreasing the oxidation rate of laser-synthesized nanomaterials [29,34].

The obtained results clearly demonstrate that nanocomposites combining Si and other chemical elements (Fe in the present study) can possess a very strong optical absorption in the visible and NIR spectral ranges, as compared to pure Si NPs. Moreover, in this study, for the first time, we measured the photothermal conversion efficiency of laser-synthesized Si-Fe NPs. The conversion coefficient was found to reach 33–42% at a 808 nm excitation radiation, which is located in the center of the window of relative transparency of biological tissues (650–950 nm). It should be noted that this value is higher than the relevant parameter for some popular nanoscale photothermal sensitizers, including Cu_9_S_5_ NPs [43] and bismuth sulfide nanorods [44], which makes the laser-ablated Si-Fe NPs a very promising candidate for potential applications in photohyperthermia treatment and photoacoustic imaging (Table 1). At the same time, the photoheating coefficients obtained for Si and Si-Fe NPs can be compared with similar values for gold nanoparticles (Au NPs) in Ref. [36]. The photothermal conversion efficiency for Au NPs is in the range of 60–80% for a 532 nm laser. If we use a laser with a wavelength near the absorption maximum for these particles, it is possible to achieve the same results as for gold NPs. Despite this opportunity, we applied laser irradiation in the transparency range of biological tissues, which corresponds better to conditions of photothermal treatment in biomedical applications. In addition, we observed the enhancement of photothermal conversion efficiency for the Si-Fe NPs compared to the Si NPs, which encourages further work on the assessment of photothermal properties of composite NPs. In particular, it would be interesting to study dependences of the photoheating efficiency on both the NP size and concentration of Fe in the composition of Si-Fe NPs, wavelength of photoexcitation, and power density of laser radiation.

In addition to important quantitative results in determining the photothermal conversion efficiency of Si-Fe NPs, two methods for determining this physical parameter were considered. A standard method to perform the task is based on the measurement of the maximal heating temperature, while an alternative approach addresses the use of the heating rate parameter at the initial stage of the time dependence. The first method allowed us to estimate that the conversion efficiency was approximately 33 ± 5%. On the other hand, the second method, based on the assessment of initial stage of the photoheating transition (i.e., the initial heating rate), provided the value of photothermal conversion efficiency of approximately 42 ± 4%. While both values are similar within the experimental error bars, the latter approach seems to be faster and more convenient to obtain the conversion efficiency, especially for semiconductor NPs (Si, Ge, Si-Fe, etc.), whose optical properties are temperature-dependent and/or are not stable during a prolonged photoheating.

The transverse proton relaxivity of laser-ablated Si-Fe NPs (*r*_2_ = 54 g^−1^s^−1^L) appears to be several times larger than that of Si-Fe NPs prepared by dry ablative synthesis in Ar plasma jets [24,25]. This fact indicates a high potential of the PLAL method to fabricate efficient NP-based contrast agents (CAs) for MRI. Considering the composition of our Si-Fe NPs, their mean molar mass is approximately 31 g, while the molar transverse relaxivity is approximately 1740 mM^−1^s^−1^. This is significantly larger than the corresponding values of clinically approved Gd-based CAs [48] and prospective CAs based on superparamagnetic iron oxide (SPIO) NPs [46,47] (Table 1). Therefore, the observed strong shortening of the transverse relaxation time, mediated by laser-synthesized Si-Fe NPs, opens up opportunities for their application as CAs for T2 contrasting.

As for the biocompatibility of S-Fe NPs, it can be noticed that similar NPs prepared in the Ar plasma jets [24,25] and by PLAL [49] demonstrate a very low level of cytotoxicity in different human cells in vitro [48]. The low toxicity of Si-Fe NPs can be explained by a low toxicity of laser-synthesized pure Si and Fe NPs [11,33].

## 5. Conclusions

Silicon–iron composite NPs were successfully synthesized by fs laser ablation in acetone, followed by their transfer to water. The synthesized aqueous solutions consisted of non-agglomerated, uniform, spherical Si-Fe NPs, while their size distribution contained two populations with the mean sizes of 20 and 90 nm. TEM and EDS studies revealed a composite structure of Si-Fe NPs, which was characterized by a nearly spatially homogeneous distribution of silicon, iron and oxygen atoms over the volume of individual NPs. Raman spectroscopy and X-Ray diffraction analysis revealed a crystalline Si phase in the composite Si-Fe NPs, as well as the presence of iron oxide and iron silicide features.

The photothermal experiments with Si-Fe NPs under irradiation with CW infrared laser (808 nm) demonstrated a superior photothermal conversion efficiency compared to the laser-ablated pure Si NPs (33–43% for Si-Fe NPs and 10–14% for Si NPs). This fact encourages a further assessment of the photothermal properties of Si-Fe NPs as a promising sensitizer of photothermal therapy for biomedical applications, as well as a contrast agent in tasks of photoacoustic imaging. Here, one has to consider the behavior of the photothermal conversion efficiency on the concentration of NPs in the suspension, the crystal structure, the photoexcitation wavelength, the power density of the laser radiation and the size of Si-Fe NPs.

Moreover, high relaxation rates of the protons (the longitudinal relaxivity of *r*_1_ = 1.7 g^−1^s^−1^L and the transverse relaxivity of *r*_2_ = 54 g^−1^s^−1^L) in the presence of Si-Fe NPs show promise for their potential theranostic applications, e.g., as CAs in MRI diagnosis and the MRI-guided photothermal therapy of tumors. Since NPs based on Si and Fe were found to be biodegradable and have low toxicity, one can expect a biodegradability option for the fabricated laser-ablated Si-Fe NPs, although this option has yet to be confirmed in further studies in vivo. We believe that the combination of optical, photothermal and MRI contrasting properties, and a high biological safety and potential biodegradability makes laser-ablated composite Si-Fe NPs a very promising multifunctional agent for cancer theranostic applications.

## Figures and Tables

**Table 1:** Comparison of photothermal and MRI properties of different NPs.

Type of NPs	Photothermal Conversion Efficiency, %	Relaxivity, mM^−1^s^−1^	Ref.
Si-Fe, laser ablated	33–43	1740 (*r*_2_)	This study
Si, laser ablated	10–14	51 (*r*_2_)	
Si-Fe from Ar plasma jets		53–820 (*r*_2_)	[24,25]
Au	60–80 (at 532 nm)		[36]
CuS	25.7		[43]
Gd-doped polymeric	26	11 (*r*_1_)	[44,45]
SPIO	20	35–200 (*r*_2_)	[46,47]

## Data Availability

The data presented in this study are available on request from the corresponding authors.

## Supplementary Materials

The following supporting information can be downloaded at: https://www.mdpi.com/article/10.3390/nano13152256/s1, Figure S1: (A) Typical TEM image of Si NPs; (B) Size distribution of Si NPs obtained from the image in panel (A), where the red curve gives a fit by the log-normal function.
