# Supplementary material for: Laser-Ablative Synthesis of Silicon–Iron Composite Nanoparticles for Theranostic Applications
Source: Nanomaterials (Basel). 2023 Aug 5;13(15):2256. doi: 10.3390/nano13152256 (PMC10421319; doi:10.3390/nano13152256)
Supplement: Supplementary file 1 [file nanomaterials-13-02256-s001.zip › nanomaterials-2470152-supplementary.pdf]

Supplementary Materials

Laser-Ablative Synthesis of Silicon–Iron Composite Nanoparticles for Theranostic Applications

Alexander A. Bubnov [1,2], Vladimir S. Belov [1], Yulia V. Kargina [1,3], Gleb V. Tikhonowski [1], Anton A. Popov [1], Alexander Yu. Kharin [1], Mikhail V. Shestakov [1,4], Alexander M. Perepukhov [5], Alexander V. Syuy [5], Valentyn S. Volkov [5], Vladimir V. Khovaylo [6], Sergey M. Klimentov [1], Andrei V. Kabashin [7,*] and Victor Yu. Timoshenko [1,3,*]

[1] Institute of Engineering Physics for Biomedicine (PhysBio), National Nuclear Research University MEPhI, 115409 Moscow, Russia; bubnovmeph@gmail.com (A.A.B.); vsbelov@mephi.ru (V.S.B.); juliakargina@gmail.com (Y.V.K.); gtikhonowski@gmail.com (G.V.T.); aapopov1@mephi.ru (A.A.P.); aykharin@mephi.ru (A.Y.K.); mvshestakov@rgau-msha.ru (M.V.S.); smklimentov@mephi.ru (S.M.K.)

[2] Endocrinology Research Centre, Dmitry Ulyanov Street 11, 292236 Moscow, Russia;

[3] Faculty of Physics, Lomonosov Moscow State University, Leninskie Gory 1, 119991 Moscow, Russia

[4] Moscow Timiryazev Agricultural Academy - Russian State Agrarian University, 127434 Moscow, Russia

[5] Moscow Institute of Physics and Technology, Dolgoprudny, 141700 Moscow Region, Russia; aleksandr-iv@mail.ru (A.M.P.); alsyuy271@gmail.com (A.V.S.); vsv.mipt@gmail.com (V.S.V.)

[6] Department of Functional Nanosystems and High-Temperature Materials, National University of Science and Technology MISIS, Leninskiy Prospekt 4, 119049 Moscow, Russia; khovaylo@misis.ru

[7] LP3, Aix Marseille University, CNRS, Campus de Luminy, Case 917, 13288 Marseille, France

* Correspondence: andrei.kabashin@univ-amu.fr (A.V.K.); timoshen@physics.msu.ru (V.Y.T.)

1. *NPs synthesis*

Crystalline silicon (c-Si) wafers were used as a target for preparation of Si NPs. There are some characteristics of wafers: resistivity of 10–20 Ohm·cm and crystallographic surface orientation (1 0 0). The surface of the wafers were covered of natural silicon oxide form. In order to remove the layer of oxide we treated wafers with an aqueous solution of HF (48%) for 1–2 s. For laser ablation we used a linearly polarized beam of a femtosecond laser (Teta 10 system, Avesta Ltd., Russia) at 1030 nm with pulse duration 270 fs, energy 100 μJ per pulse, and repetition rate 10 kHz. A laser beam (3 mm in diameter) was focused onto target immersed in 10 mL of deionized water at normal incidence. The laser synthesis was done for 1 h at room temperature. Aqueous suspensions of laser-synthesized Si NPs with initial concentration of 0.1 mg/mL were centrifuged (12000 g, 20 min) to obtain the concentration of 4 mg/mL. In further experiments the suspension was diluted to get the concentration about 1 and 0.1 mg/mL to study the photoheating and optical extinction, respectively.

2. *TEM analysis*

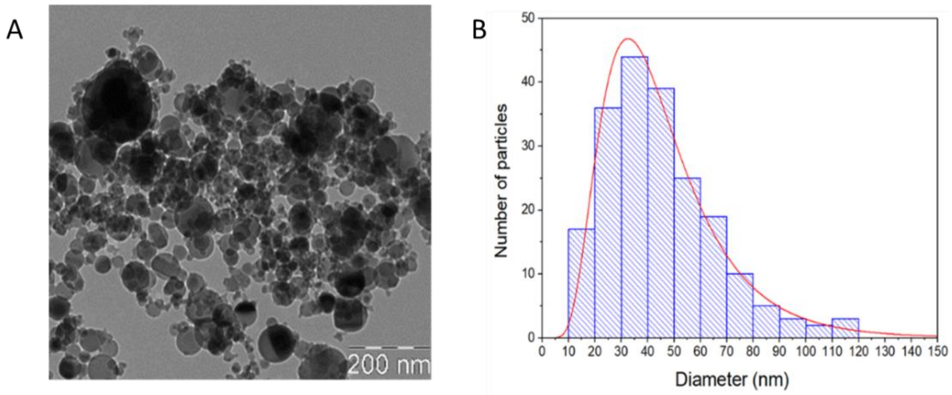

Figure S1. (A) Typical TEM image of Si NPs; (B) Size distribution of Si NPs obtained from the image in panel A where the red curve gives a fit by the lognormal function.
